# Lateral flow assay for rapid serodiagnosis of bovine leptospirosis

**DOI:** 10.22099/IJVR.2021.41151.5974

**Published:** 2022

**Authors:** K. Senthilkumar, G. Ravikumar

**Affiliations:** Zoonoses Research Laboratory, Centre for Animal Health Studies, Tamil Nadu Veterinary and Animal Sciences University, Chennai 600051, Tamil Nadu, India

**Keywords:** Bovine, Immunochromatography, Lateral flow assay, Leptospirosis

## Abstract

**Background:**

Leptospirosis is considered to be an economically important disease in bovine. The disease burden is not appropriately monitored due to cumbersome serological tests that could be performed only in established laboratories. This warrants the development of a field level rapid diagnostic test.

**Aims:**

The study aimed to develop a lateral flow assay (LFA)-based pen-side diagnostic test to detect antibodies to *Leptospira*.

**Methods:**

LFA strip was prepared with the heat extracted antigen from *L. interrogans* serovar Pomona. To assess the performance of the developed LFA, a total of 300 bovine serum samples with their clinical histories were used and the initial screening for *Leptospira* antibodies was performed by the standard microscopic agglutination test (MAT). The sensitivity, specificity, and agreement (kappa value) were calculated between developed LFA and MAT. The stability of LFA was evaluated on days 30, 60, 90, and 120.

**Results:**

Out of 300 samples tested, 225 were positive, and 75 were negative on MAT and 208 were positive, and 92 were negative on LFA. The developed LFA had a sensitivity of 90.7% and a specificity of 94.7%. The results of the assay were substantially in agreement with MAT, with a kappa value of 0.79. The LFA strips were stable for 120 days at 4°C.

**Conclusion:**

A Lateral flow assay-based rapid pen-side test was developed and its utility to diagnose bovine leptospirosis was evaluated.

## Introduction

 Leptospirosis is a widespread bacterial zoonosis with increasing importance due to its disease severity and economic loss to the agrarian community. The disease is mainly transmitted from the contaminated environments and carrier animals. The genus *Leptospira* comprises 66 genomospecies with 25 serogroups and more than 300 pathogenic serovars (Caimi and Ruybal, 2020). It is known to affect more than 160 mammalian species. The studies on the seroprevalence of bovine leptospirosis in India are estimated to vary from 10.1% to 42.1% (Srivastava et al., 1983; Biswal et al., 2000; Rani Prameela et al., 2013; Jai sunder et al., 2018; Senthilkumar et al., 2021). Bovine leptospirosis is often subclinical rather than the clinical form with the signs of high-temperature (103° to 105° F), marked drop in milk yield, highly colored yellowish urine, and icteric mucous membrane (Radostits et al., 2010). Early diagnosis and appropriate treatment with robust antibiotics would reduce the production loss in bovine. Since the animals are the reservoir hosts, studies on the disease prevalence in animals are important to implement control measures as well as to prevent zoonoses.

 With respect to the diagnosis of leptospirosis, dark-field microscopy and serological tests are routinely preferred. The detection and confirmation of leptospires by culture and isolation, take 2-8 weeks, and does not fulfill the requirement of a veterinarian for treatment and implementing appropriate control measures. Among the serological tests, microscopic agglutination test (Dikken and Kmety, 1978), enzyme-linked immunosorbent assay (Terpstra et al., 1985), immunofluorescence-antibody test (Appassakij et al., 1995), Lepto dipstick test (Gussenhoven et al., 1997), indirect hemagglutination test (Levett and Whittington, 1998), and Latex agglutination test (Behera et al., 2021) have been used. The gold standard microscopic agglutination test (MAT) requires an established well-equipped laboratory that maintains several *Leptospira* reference strains to be used as antigens and the technical expertise to read and interpret the results. The MAT titres are usually lower during the acute stage of the disease, hence, diagnosis based on a single serum sample is difficult and needs testing of paired sera samples (Faine, 1982). The IgM ELISA detects the antibodies at the end of the first week of illness (Terpstra et al., 1985), however, the limited shelf-life of reagents and the requirement of ELISA readers limit it in poor resource settings. The serological test based Lepto dipstick overcame these problems but required more incubation times for reading the results (Gussenhoven et al., 1997). To overcome these pitfalls, the Royal Tropical Institute (KIT), Amsterdam, Netherlands has developed a diagnostic test based on lateral flow and applied it for rapid diagnosis of human leptospirosis (Smits et al., 2001). In this study, a similar lateral flow assay-based diagnostic test was developed to detect antibodies against *Leptospira* in bovines, and its efficacy in terms of diagnostic sensitivity, specificity, and accuracy to the widely applied MAT was also assessed.

## Materials and Methods

**Clinical samples and screening by microscopic agglutination test (MAT)**

 A total of 300 blood samples were collected from cattle from different agro-climatic zones of Tamil Nadu (North-East zone, Northwest zone, West zone, Cauvery Delta, South zone, Hilly region, and High rainfall region), and their serums were separated and stored at -20°C. These animals had a clinical history of fever (102), abortion (10), mastitis (60), jaundice (12), infertility (26), and healthy (90). A panel of twelve *Leptospira* reference strains (Table 1) was used for the microscopic agglutination test that is kept in Ellinghausen-McCullough-Johnson-Harris (EMJH) medium (Difco Laboratories, USA) at the Zoonoses Research Laboratory, Tamil Nadu Veterinary and Animal Sciences University, Chennai, Tamil Nadu. The serum samples were initially screened for anti-leptospiral antibodies, showing leptospiral infection, and the MAT titer of 1:100 and above was considered as positive (OIE, 2018).

**Preparation of the heat extracted antigen**

 The *L. interrogans* serovar Pomona culture with a density of ~ 2 × 10^8^ leptospires/ml was pelleted and washed thrice with phosphate buffered saline (PBS) and the final cell pellet was re-suspended in bicarbonate buffer (pH = 9.6). The suspension was denatured by incubating in a boiling water bath for 30 min, centrifuged at 10,400 g for 30 min to remove cell debris, and the supernatant was concentrated by filtering through a 10 kDa concentrator. The protein concentration of the retentate was determined using the micro bicinchoninic acid (BCA) kit (M/s Thermo Scientific, USA), then, aliquoted, and stored at -20°C. This denatured and concentrated total protein was used as the antigen in the design of the lateral flow assay.

**Preparation of the lateral flow assay strip**

 The nitrocellulose membrane strip (M/s Advanced Microdevices Pvt Ltd., India) flanked at one end with the colloidal gold protein A conjugate pad and the other with the absorption pad was used as a laminate. The heat extracted antigen and anti-bovine IgG were printed as test and control lines, respectively on the laminate using an Easy printer (M/s Advanced Microdevices Pvt Ltd., India). The laminate was cut into 3 mm strips using a programmable strip cutter to fit into a plastic cassette and was used as the individual test strip. The assay was performed by adding test serum (10 µL) on the sample pad followed by the sample buffer (70 µL), consisting of phosphate buffered saline with 0.66 mg of bovine serum albumin per ml and 3% Tween 20 (Smits et al., 2001). This resulted in the migration of the test sample into the LFA strip; the test was considered as valid on the development of coloured (pink) lines on both tests and control lines with the positive control (anti-*Leptospira* hyperimmune sera raised in rabbits) and only on the control line with the negative control sample (*Brucella* sp. anti-serum).

**Screening field sera samples with the LFA assay**

 The 300 bovine serum samples were tested by lateral flow assay that were seroreactive to different MAT serogroups, ranging from 1:100 to 1:3200 titers, and negative (Table 1). The sensitivity, specificity (as measures of validity), and agreement (kappa values) between the developed LFA and the MAT assay were calculated using Chi-square test and Kappa statistics (M/s GraphPad software). The accuracy, positive predictive value, and negative predictive value were also calculated for the developed LFA test (M/s MedCalc

**Table 1:** Serogroup reactivity of bovine sera on microscopic agglutination test and lateral flow assay

Microscopic agglutination test				Lateral flow assay
Serogroup	Serovar	Strain	No of positives	
Australis	Australis	Ballico	51	
Autumnalis	Rachmati	Rachmati	7	
Ballum	Ballum	Mus 127	2	
Canicola	Canicola	Hond Utrecht IV	4	
Grippotyphosa	Grippotyphosa	Moskva V	5	
Sejroe	Hardjo	Hardjoprajitno	67	
Hebdomadis	Hebdomadis	Hebdomadis	26	
Icterohaemorrhagiae	Icterohaemorrhagiae	RGA	20	
Javanica	Poi	Poi	13	
Pomona	Pomona	Pomona	14	
Pyrogenes	Pyrogenes	Salinem	11	
Tarassovi	Tarassovi	Perepelitsin	5	
**Total positive**			225	208
**Total negative**			75	92

software). The lateral flow assay strips were kept in a moisture-resistant sachet and stored at 4°C in vacuum desiccators. The stability was assessed on days 30, 60, 90 and 120 with known positive and negative serum samples.

## Results

 The antigen concentration of 200 µg/ml (from the heated extract of* L*. *interrogans *serovar Pomona) and the rabbit anti-bovine IgG concentration of 1 mg/ml were found optimum for test and control lines in the LFA strip. The protein A gold conjugate with three OD dilution was found to be optimum. Out of 300 serum samples tested by MAT, 225 samples showed seroreactivity to different serogroups of *Leptospira* with antibody titers ranging from 1:100 to 1:3200. The serogroup showing the high titer was considered as the infecting serogroup. The results are detailed in Tables 1 and 2.

**Table 2:** Seroreactivity of sera at different dilutions on microscopic agglutination test and lateral flow assay

Microscopic agglutination test			Lateral flow assay	
Titre	Positive	Negative	Positive	Negative
Nil	-	75	4	71
1:100	51	-	34	17
1:200	71	-	67	4
1:400	62	-	62	-
1:800	20	-	20	-
1:1600	15	-	15	-
1:3200	6	-	6	-
Total	225	75	208	92

 Out of 300 serum samples tested by LFA, 208 and 92 samples respectively showed positive and negative reactions (Tables 1 and 2). The sensitivity of LFA was 90.7% (95% CI: 86.1% to 94.1%), and the specificity was 94.7% (95% CI: 86.9% to 98.5%) (Table 3). The kappa value of 0.79 (95% CI: 0.72 to 0.87) indicated substantial agreement between the lateral flow assay and the microscopic agglutination test. LFA showed 91.7% accuracy of diagnosis with a positive predictive value of 98.1% and a negative predictive value of 77.2%, indicating a rapid diagnostic test in endemic areas. The sensitivity of LFA increased from 66.6% in samples with the MAT titer of 1:100 to 94.3% in samples with 1:200, and 100% in samples with MAT titer of 1:400 and above. The LFA strips when stored at 4°C in vacuum desiccators were found to be stable until 120 days.

## Discussion

 The laboratory diagnosis of bovine leptospirosis mainly relies on the detection of serogroup-specific antibodies by microscopic agglutination test. The limitations of this method include the requirements for an elaborate laboratory setup, the complexity of the assay procedure, the requirement for the maintenance of the reference leptospiral serovars, and expertise to visualize the results. It is also challenging to recognize leptospirosis by a single MAT titer in the active infection or patients who have low MAT titers. MAT has also been reported to give false-positive results (a titer of 1:80 or 1:100) due to cross-reactive antibodies in brucellosis, salmonellosis, rheumatoid fever, and lyme disease. This is usually overcome by demonstrating the raising titers in paired sera samples which has a diagnostic significance, but it further delays the disease diagnosis. Hence, a rapid, point-of-care test tailor-made for large-scale screening of sera samples in endemic areas without using any sophisticated equipment is always preferred. The field applicable pen side test such as Lepto Dipstick had been evaluated for rapid serodiagnosis of leptospirosis but it had low sensitivity (Gussenhoven et al., 1997). The alternative test, the immunochromatography-based lateral flow assay had been evaluated as a rapid, point of care diagnostic test for human leptospirosis (Smits et al., 2001; Vanithamani et al., 2015; Doungchawee et al., 2017; Maze et al., 2019).

 The whole-cell heat extracted antigen from pathogenic *Leptospira* was used for the development of LFA in this study. A similar type of heat resistant antigen from non-pathogenic leptospiral strain (strain Patoc I) was used to develop a lateral flow assay kit for human leptospirosis (Smits et al., 2001; Maze et al., 2019). The other type of antigens used for the lateral flow assay included lipopolysaccharide (LPS) (Priya et al., 2003; Doungchawee et al., 2017), and sonicated whole antigen for dot-ELISA (Tansuphasiri et al., 2005). The extraction of LPS is cumbersome and time-consuming, and the preparation of the sonicated antigen needs a sonicator; hence, heat extracted antigen from the whole cell was preferred as a rapid and simple method.

 The prepared lateral flow assay produced two stained lines (pink colour) in the positive reaction and one line in the negative reaction, consistent with the lateral flow assay developed for human leptospirosis, and this was included all days to determine the validity of the assay developed (Smits et al., 2001; Sehgal et al., 2003). The sensitivity (90.7%) and specificity (94.7%) of lateral flow assay for bovine leptospirosis in this study are comparable with the sensitivity (85.8%) and specificity (93.6%) of lateral flow assay for human leptospirosis (Smits et al., 2001). The results of LFA in detecting sera samples with MAT titer of 1:400 and above were consistent. Hence, it is applicable for the diagnosis of leptospirosis at this MAT titer and is defined as diagnostic criteria for leptospira infection for a single serum sample (Vijayachari, 2007). The low sensitivity of LFA with the samples of 1:100 MAT titer could be attributed to the cross-reacting antibodies to other infectious agents as indicated in earlier reports. This can be overcome by testing the paired sera samples from clinically suspected animals.

**Table 3:** Calculation of relative sensitivity and specificity of lateral flow assay for detection of anti-leptospiral antibodies in bovine sera in comparison with MAT

Diagnostic test		Microscopic agglutination test result (Nos)		
		Positive	Negative	Total
Lateral flow assay result (Nos)	Positive	204	4	208
	Negative	21	71	92
	Total	225	75	300

Sensitivity: True positive/true positive + false negative (204/225=90.7%), and specificity: True negative/true negative + false positive (71/75=94.7%)

 The results of the developed lateral flow assay are substantially in agreement with MAT with a kappa value of 0.79 and this correlates with the report of Sehgal et al. (2003) with a kappa value of 0.74 during 2-4 weeks of illness screened with the Lepto lateral flow kits supplied by Royal Tropical Institute, The Netherlands. This indicates the suitability of LFA to specifically confirm *Leptospira* infection in endemic areas. The production of the same type of result indicated the stability of the LFA strips at 4°C for 120 days and is applicable at the field level. This is in agreement with the earlier report that the strips were stable without loss of reactivity for a prolonged period (Smits et al., 2001). The rapid, simple, ease of use, and comparable results, imply that the developed lateral flow assay can be used at the field level as an alternative to MAT for diagnosing bovine leptospirosis. Further studies are required to improve the sensitivity and specificity of the assay and also screening the sera samples for the other cross-reacting antibodies.

 Early diagnosis of bovine leptospirosis will enable the early application of treatment and prevention approaches. The lateral flow assay developed in this study is easy to use and the results are visible in two minutes. It has a sensitivity of 90.7% and a specificity of 94.7% and correlates well with the gold standard microscopic agglutination test. The lateral flow assay strips are stable, without loss of reactivity for a prolonged period (120 days). The positive predictive value of 98.1% indicated the suitability of the test for confirmation of disease; however, further study is required to improve the assay’s sensitivity to be used as a screening test at the field level.

## Conflict of interest

 The authors declared no conflict of interest.
